# Construing Morality at High versus Low Levels Induces Better Self-control, Leading to Moral Acts

**DOI:** 10.3389/fpsyg.2017.01041

**Published:** 2017-06-21

**Authors:** Chia-Chun Wu, Wen-Hsiung Wu, Wen-Bin Chiou

**Affiliations:** ^1^Institute of Education, National Sun Yat-sen UniversityKaohsiung, Taiwan; ^2^Department of Healthcare Administration and Medical Informatics, Kaohsiung Medical UniversityKaohsiung, Taiwan

**Keywords:** construal levels, honesty, morality, self-control, volunteerism

## Abstract

Human morality entails a typical self-control dilemma in which one must conform to moral rules or socially desirable norms while exerting control over amoral, selfish impulses. Extant research regarding the connection between self-control and level of construal suggest that, compared with a low-level, concrete construal (highlighting means and resources, e.g., answering ‘how’ questions), a high-level, abstract construal (highlighting central goals, e.g., answering ‘why’ questions) promotes self-control. Hence, construing morality at higher levels rather than lower levels should engender greater self-control and, it follows, promote a tendency to perform moral acts. We conducted two experiments to show that answering “why” (high-level construal) vs. “how” (low-level construal) questions regarding morality was associated with a situational state of greater self-control, as indexed by less Stroop interference in the Stroop color-naming task (Experiments 1 and 2). Participants exposed to “why” questions regarding morality displayed a greater inclination for volunteerism (Experiment 1), showed a lower tendency toward selfishness in a dictator game (Experiment 2), and were more likely to return undeserved money (Experiment 2) compared with participants exposed to “how” questions regarding morality. In both experiments, self-control mediated the effect of a high-level construal of morality on dependent measures. The current research constitutes a new approach to promoting prosociality and moral education. Reminding people to think abstractly about human morality may help them to generate better control over the temptation to benefit from unethical acts and make it more likely that they will act morally.

## Introduction

In principle, self-control is defined as the ability to regulate one’s own thoughts, emotions, impulses and behavior (Ainslie, 1975; Baumeister et al., 2007). Self-control has been shown to play an important role in focusing on superordinate, distant goals while resisting the temptation of smaller, proximal reward and gratification (Mischel et al., 1989; Myrseth and Fishbach, 2009; Fujita and Sasota, 2011). The term “construal" refers to the mental representation of an event or object (Trope and Liberman, 2003, 2010). Recent advancements in the connection between construal level and self-control suggest that engaging in abstract, global (high-level) construal is more likely to promote self-control success, compared with engaging in concrete, local (low-level) construal (Freitas et al., 2004; Fujita et al., 2006a; Agrawal and Wan, 2009; Schmeichel and Vohs, 2009; Fujita and Carnevale, 2012; Chiou et al., 2013; Chang and Chiou, 2015). Thus, construing morality at higher levels should boost self-control and, thereby, enhance moral behavior. In this article, we provide experimental evidence showing that a brief mindset-based intervention promotes the tendency to act morally. This is the first study showing that construing human morality at high levels may lead to a greater tendency to prosociality and honesty.

### Construal Levels and Self-control

In general, regulating thoughts or emotions, resisting temptation, and maintaining good self-discipline all require the exertion of self-control (Fujita, 2011). Construal level theory (CLT) posits that individuals can mentally represent the same event or object at high or low construal levels (Trope and Liberman, 2003). CLT proposes that high-level construal is relatively abstract and a superordinate mental representation compared with low-level construal (Trope and Liberman, 2010). For example, an “Apple iPhone 7” can be represented (i.e., an object) as a “cellular phone” (concrete, low-level construal) or as a “communication device” (abstract, high-level construal). Similarly, “attending a family reunion” (i.e., an event) can be construed as “respecting tradition” (abstract, high-level construal) or “going to a restaurant” (concrete, low-level construal).

According to CLT (Trope and Liberman, 2010), a high-level construal mindset may lead people to appreciate the superordinate, distal goals of their choices and thereby enhance their resistance to proximal temptation (i.e., a state of better self-control). In contrast, a low-level construal mindset may direct attention toward subordinate, salient features of their choices and thereby reduce the ability to defer immediate gratification (i.e., self-control failure; Fujita et al., 2006a). Recent studies have demonstrated the effect of construal levels on self-control-related behaviors (e.g., Freitas et al., 2004; Fujita et al., 2006b; Chang and Chiou, 2015). In pioneering research on mindset-based manipulation of the construal level, Liberman and Trope (1998) conducted a series of studies showing that events construed at higher levels focus more on goals, or “why” aspects, whereas events construed at lower levels focus on means, or “how” aspects of the event. Similarly, Freitas et al. (2004) induced participants to consider either “why” or “how” they engage in a behavior (e.g., maintaining good physical health). They found that, compared with participants in a low-level construal mindset, those in a high-level construal were faster to list distant goals than immediate emotional reactions. Using the why/how paradigm to manipulate construal levels, Fujita et al. (2006b) demonstrated that participants with high relative to low construal levels exhibit greater self-control. Prior research regarding the depletion of self-control has shown that participants induced to adopt low-level construal mindsets show poor self-control, but those induced to adopt high-level construal mindsets do not show the typical depletion pattern (Agrawal and Wan, 2009; Schmeichel and Vohs, 2009). Furthermore, an abstract, high-level (relative to a concrete, low-level) construal mindset is associated with a more negative attitude toward temptations (Fujita and Han, 2009) and lower discounting rates (Malkoc et al., 2010), suggesting greater self-control. Smokers adopting a high-level (relative to a low-level) construal of physical health exhibit better control over the impulse to smoke cigarettes (Chiou et al., 2013). A high-level construal (goal-focused; ‘why’ aspects) mindset regarding weight loss led to greater control over dietary practice compared with a low-level construal (means-focused: “how” aspects) mindset (Chang and Chiou, 2015).

According to the notion of CLT, adopting a high-level construal mindset can engender a focus on central, distal goals while overlooking salient, proximal temptations. Hence, high-level construal may promote the capacity to delay gratification (i.e., a manifestation of better self-control). Research conducted by Fujita and Sasota (2011) also suggested that construing an event at a high level promotes the cognitive accessibility of remote goals while expensing immediate temptations, leading to greater self-control. Given that activation of high-level construal rather than low-level construal may induce better self-control (see Fujita and Carnevale, 2012, for a related review), we argue that a state of greater self-control should be induced by a high-level construal mindset than a low-level construal mindset.

### Self-control and Moral Behavior

Based on the strength model of self-control (Baumeister et al., 2007), morality entails a typical self-control dilemma in which one must conform to moral rules while exerting control over amoral, selfish impulses (Carver and Scheier, 1981; Baumeister et al., 1994). Self-control has been referred to as “moral muscle,” corresponding to the ability to override selfish, antisocial impulses in favor of socially desirable norms (Baumeister and Exline, 1999). Individuals must overcome prepotent, selfish impulses before acting morally (DeWall et al., 2008; Steinbeis et al., 2012). In other words, to act morally or prosocially, people should exert self-control to suppress unwanted behavior (Stevens and Hauser, 2004). Evidence showing that low self-control plays a key role in criminal or antinormative behaviors supports the importance of self-control in human morality (Gottfredson and Hirschi, 1990; Hirschi, 2004). Therefore, greater self-control should be associated with a greater tendency to act morally or ethically (Baumeister and Exline, 1999; Hirschi, 2004; Mead et al., 2009).

Mead et al. (2009) demonstrated that participants with lower self-control exaggerated their performance for monetary gain in a self-scored test (i.e., dishonesty; Experiment 1) and showed greater susceptibility to cheating (Experiment 2) than their non-depleted counterparts. Chiou et al. (in press) showed that a situational state of low self-control is associated with a decreased tendency to return undeserved money (Experiment 2) and an increased tendency to cheat in a matrix task (Experiments 3 and 4). Additionally, neuroimaging evidence has shown that impulse control involves control-related regions in the prefrontal cortex (Baumgartner et al., 2011; Harris et al., 2013; Hare et al., 2014). Given that better self-control is associated with increased morality, we hypothesized that construing morality at high versus low levels should engender greater self-control, leading to a stronger tendency to perform moral acts.

### The Present Research

Two experiments were conducted to test whether construing morality at higher levels (the “why” paradigm; e.g., Freitas et al., 2004; Fujita et al., 2006b; Chiou et al., 2013) could be linked with greater state self-control, as indexed by smaller Stroop interference in the Stroop color-naming task (e.g., von Hippel and Gonsalkorale, 2005; Gailliot et al., 2007; Carr and Steele, 2010; Chiou et al., 2013; Wu et al., 2017), compared with construing morality at lower levels. Experiment 1 examined the connection between answering “why” (high-level construal) or “how” (low-level construal) questions regarding morality and subsequent volunteer behavior (i.e., volunteerism; Vohs et al., 2006; Chiou and Cheng, 2013). Experiment 2 examined whether participants in a high-level construal mindset of morality (vs. a low-level construal mindset of morality and a control condition) would show less selfishness in a dictator game (i.e., altruism; Hoffman et al., 1994; Shariff and Norenzayan, 2007; Chiou and Cheng, 2013) and would be more likely to return undeserved money (i.e., honesty; Chiou and Cheng, 2013; Yap et al., 2013; Chiou et al., in press). The hypothetical mechanism (i.e., state self-control) underlying the relationship between construing morality at a high-level of construal and a greater tendency to perform moral acts was tested in Experiments 1 and 2.

## Experiment 1: Construal Levels, Self-Control, And Volunteerism

### Method

#### Participants

In total, 102 undergraduates and graduate students (48 females, 54 males; mean age = 20.6 years, *SD* = 2.1) at a private university in southern Taiwan were recruited to participate in this experiment via campus posters. The sample size was estimated by calculating the number of participants required to test a directional hypothesis regarding the mean difference between two independent groups under the following conditions: α = 0.05, *d* = 0.50 (medium effect size; Cohen, 1988) and power = 0.80.

#### Procedure

Upon arrival, participants were informed that they would engage in unrelated tasks. They were further told that these tasks would be used in future research on cognitive evaluation. After providing written consent, the participants were randomly assigned to receive either a high-level construal or low-level construal intervention. The present research employed the why/how paradigm to manipulate high/low level construal. Participants were instructed to answer a four-layer ladder questionnaire about “human morality.” The experimental task took approximately 6–10 min to complete. Prior studies have demonstrated that answering “why” questions is effective in inducing a high-level construal mindset, whereas answering “how” questions is effective in inducing a low-level construal mindset (e.g., Liberman and Trope, 1998; Freitas et al., 2004; Fujita et al., 2006b; Chiou et al., 2013; Chang and Chiou, 2015).

Following the why/how paradigm (Freitas et al., 2004), participants under the high-level construal condition started at the bottom of the ladder and moved up, generating increasingly superordinate answers to the question of “why” they would act morally (e.g., maintain a positive self-image, build a better tomorrow). Specifically, if an answer to the first-rung “why” question were “maintain a positive self-image,” the participant then answered “Why would you maintain a positive self-image” in response the second-rung question. The question at the next rung of ladder depends on the answer to the question at the prior rung. Participants under the low-level construal condition moved down the ladder, generating increasingly subordinate answers to the question of “how” they would act morally (e.g., reminding myself of role models, reminders of ethical codes). For example, if an answer to the first rung of the “how” question were “reminding myself of role models,” the participant then answered “How would you remind yourself of role models” as the second-rung question. Accordingly, by inducing all participants to think about moral acts, the construal focus was kept the same while varying the abstraction level.

The construal-level task was followed by the computerized Stroop task, which is one of the most widely used measures of state self-control (e.g., Gailliot et al., 2007; Carr and Steele, 2010; Chiou et al., 2013, in press; Wu et al., 2017). The Stroop color-naming task requires the respondent to name the font color of a series of color words. When a color word is inconsistent with its font color (i.e., an incongruent trial), a correct color-naming response requires the exercise of self-control to ignore the word’s meaning. Therefore, the Stroop color-naming task may represent a useful index of state self-control (Long and Prat, 2002; Richeson and Shelton, 2003). On each trial, a color word is displayed in a font color that is either congruent (e.g., the word *blue* in blue font) or incongruent (e.g., the word *blue* in red font) with the word’s meaning. After six practice trials (all congruent), participants were presented with 16 congruent trials and 16 incongruent trials in randomized order, with a string of *X*s displayed for 500 ms between trials. Participants were instructed to report the font color of each word as quickly as possible by pressing color-coded keys.

Later, participants completed demographic questions and the Positive and Negative Affect Schedule (Watson et al., 1988; positive affect: ranged from 1 to 5, α = 0.84; negative affect: ranged from 1 to 5, α = 0.83). At the end of the experiment, the experimenter who is a graduate student entered the test room and claimed that she was looking for help coding data. Participants were further told that it requires approximately 5 min to code each data sheet. They were then left alone to provide their contact information and indicated how many sheets, if any, they would be able to help (Chiou and Cheng, 2013). The number of data sheets they volunteered to code was the dependent measure.

### Results

With respect to manipulation check, two blinded judges coded each participant’s responses based on the abstractness of responses: -1 = a subordinate means; +1 = a superordinate end; and 0 = a response fits neither criterion (Liberman and Trope, 1998; Freitas et al., 2004; Chiou et al., 2013; Chang and Chiou, 2015). Ratings of each participant’s four responses were then averaged to create an index of level of construal ranging from -4 to +4. The inter-judge agreement on averaged ratings was good (*r* = 0.895, *p* < 0.001). Higher scores indicate higher construal levels. As expected, participants exposed to ‘why’ questions (*M* = 3.14, *SD* = 0.78) generated responses that reflected higher levels of construal compared with those exposed to ‘how’ questions (*M* = -3.07, *SD* = 1.27) [*t*_(100)_ = 29.738, *p* < 0.001, Cohen’s *d* = 5.89].

Positive affect (PA) and negative affect (NA) were not related to the self-control measure (PA: *r* = -0.032, *p* = 0.749; NA: *r* = 0.102, *p* = 0.309) and the number of data sheets volunteered to code (PA: *r* = 0.132, *p* = 0.186; NA: *r* = -0.161, *p* = 0.105). Both PA [*t*_(100)_ = -0.647, *p* = 0.519] an NA [*t*_(100)_ = 1.525, *p* = 0.13] did not differ between the two study conditions (**Table 1**). Therefore, they were not used as covariates in subsequent analyses.

**Table 1:** Descriptive statistics for the measures in Experiment 1.

Measure	Low-level construal		High-level construal		*t*-Value
	*M*	*SD*	*M*	*SD*	
Positive affect (1–5)	2.50	0.48	2.56	0.53	-0.647
Negative affect (1–5)	1.68	0.35	1.58	0.37	1.525
Mean RT in incongruent trials (ms)	803.88	157.76	726.14	144.74	2.593^∗^
Mean RT in congruent trials (ms)	667.90	138.11	628.90	126.55	1.487
Self-control measure (ms)	138.98	46.75	97.24	40.61	4.468^∗^
Number of data sheets volunteered to code	5.24	3.08	7.12	3.13	-3.064^∗^

*Each condition involved 51 participants. The self-control measure was calculated as the mean difference in reaction time between incongruent and congruent trials in the Stroop task. Less Stroop interference indicates greater state self-control. Asterisks indicate significant mean differences between the two conditions (*p* < 0.05)*.

We employed the Stroop task to assess state self-control. Incorrect trials (<2.0%) were excluded from the analysis. State self-control was indexed by the mean difference in the reaction time (RT) between incongruent and congruent trials (i.e., Stroop interference; Gailliot et al., 2007). Greater Stroop interference indicates a lower state of self-control, suggesting that a reduced capacity for self-control results in a longer time to name the font color. As shown in **Table 1**, there were no significant differences between conditions in the mean RT in congruent trials of the Stroop task [*t*_(100)_ = 1.487, *p* = 0.14], but the mean RT in incongruent trials was longer under the low-level construal than under the high-level construal condition [*t*_(100)_ = 2.593, *p* = 0.011, *d* = 0.51]. Furthermore, Stroop interference (i.e., the self-control measure) was significantly less under the high-level construal condition than under the low-level construal condition [*t*_(100)_ = -4.468, *p* < 0.001, *d* = 0.95]. The effect of condition on state self-control did not interact with the participant sex [*F*_(1,98)_ = 0.155, *p* = 0.695]. More importantly, participants exposed to ‘why’ questions (high-level construal) volunteered to help code more data sheets than did those exposed to ‘how’ questions (low-level construal) [*t*_(100)_ = 3.064, *p* = 0.003, *d* = 0.61]. Additionally, this experimental effect did not interact with the participant sex [*F*_(1,98)_ = 0.736, *p* = 0.393].

**FIGURE 1:**
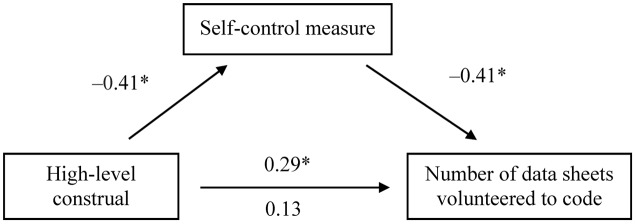
Mediation of the effect of exposure to “why” questions (high-level construal) regarding morality on the number of data sheets participants volunteered to code in Experiment 1. Values are standardized regression coefficients. The self-control measure was indexed as the mean difference in reaction time (ms) between incongruent and congruent trials in the Stroop task. Less Stroop interference indicates a state of greater self-control. On the lower path, the values below and above the arrow are the results of analyses in which the mediator was and was not included in the model, respectively. Asterisks indicate significant results (*p* < 0.01).

We employed linear regression to examine whether state self-control mediated the effect of construal levels on the number of data sheets participants volunteered to code, treating the low-level construal condition as the reference category (1 = high-level construal, 0 = low-level construal). Under the high-level construal condition, participants showed less Stroop interference [i.e., greater self-control; *B* = -38.75, *SE* = 8.67, *t*_(100)_ = -4.468, *p* < 0.001], and less Stroop interference predicted the number of data sheets volunteered to code [*B* = -0.03, *SE* = 0.01, *t*_(100)_ = -5.231, *p* < 0.001]. When the self-control measure and condition were both included as predictors of the number of date sheets volunteered to code, the self-control measure remained significant [*B* = -0.03, *SE* = 0.01, *t*_(99)_ = -4.266, *p* < 0.001], but condition did not [*B* = 0.80, *SE* = 0.62, *t*_(99)_ = 1.288, *p* = 0.201; **Figure 1**]. A bootstrap analysis (Preacher and Hayes, 2004) showed that the 95% bias-corrected confidence interval [CI: 0.42–1.99] for the indirect effect (*B* = 1.08, *SE* = 0.40; bootstrap resamples = 5,000) excluded zero, suggesting significant mediation.

Additionally, we tested whether the number of date sheets volunteered to code would mediate the connection between the construal manipulation and state self-control. The dummy variable (1 = high-level construal, 0 = low-level construal) was used in the alternative model. A high-level construal mindset toward morality predicted the number of date sheets volunteered to code [*B* = 1.88, *SE* = 0.61, *t*_(100)_ = 3.064, *p* = 0.003] and the number of date sheets volunteered to code predicted the self-control measure [*B* = -5.56, *SE* = 1.30, *t*_(100)_ = -4.266, *p* < 0.001]. However, the relationship between engagement in high-level construals and less Stroop interference remained significant [*B* = -28.28, *SE* = 8.38, *t*_(99)_ = -3.375, *p* = 0.001] when we controlled for the number of date sheets volunteered to code. These results indicate that the number of date sheets volunteered to code might not be a major mediator between construal level and state self-control.

### Discussion

The results of our first experiment replicate previous findings showing that adopting a high-level construal mindset can lead to better self-control than did adopting a low-level construal mindset (Freitas et al., 2004; Fujita et al., 2006b; Chiou et al., 2013; Chang and Chiou, 2015). This study supports the link between level of construal and moral behavior by showing that compared to answering ‘how’ questions regarding morality, answering ‘why’ questions can engender greater self-control, lead to an increased tendency toward volunteerism. Given that Experiment 1 only involved interventions of low-level and high-level construals, a non-intervention control condition which may provide the baseline assessment of dependent measures was included in the next experiment.

## Experiment 2: High-Level Construal is Associated with Less Selfishness and Increased Honesty

### Method

#### Participants

A total of 90 undergraduate students (42 females and 48 males; mean age = 20.9, *SD* = 1.4) enrolled at a public university in southern Taiwan participated in this experiment for extra course credit. The sample size was determined by calculating the number of participants required to satisfy the omnibus *F*-test (number of groups = 3) under the following conditions: α = 0.05; ω^2^ = 0.10; and power (1 – β) = 0.80 (Kirk, 2012, p. 925).

#### Procedure

Upon arrival in the laboratory, participants were informed that they were helping us test several experimental tasks that would be used in future research. After providing written consent, every three same-sex participants were randomly assigned to one of the three between-subjects experimental conditions (high-level construal, low-level construal, and control) by using a block randomization schedule. The proportions of the sexes were identical in the three study conditions. The construal-level manipulation was identical to that used in Experiment 1. Participants under the high-level construal condition answered “why” questions regarding acting morally, whereas those under the low-level construal condition answered “how” questions. Control participants did not receive experimental manipulation.

All participants performed the same computerized Stroop task used in Experiment 1. Subsequently, each participant played a one-shot, anonymous version of the dictator game (Hoffman et al., 1994), which has been widely used to measure selfishness and altruism (e.g., Shariff and Norenzayan, 2007; Chiou and Cheng, 2013). Participants were led to believe that they had been randomly paired with another person in a different room. Participants were told: “This game includes two roles: initiator and recipient. The initiator has NT $160 to allocate between him/herself and the recipient. Initiators keep whatever they do not offer to the recipients. Recipients can choose to accept or reject the offer, but their choices do not affect the initiator’s outcomes.” Although participants were told they had been randomly assigned to a role, all served as the initiator and played against the experimenter via a computer program.

After the participants made the decisions, payment in the amount that participants kept for themselves was given to participants in unsealed envelopes. The experimenter asked participants to make sure they had received the payment they deserved and exited the room. However, each participant received additional money (one NT $50 coin; Chiou and Cheng, 2013). Whether or not participants returned this undeserved, excess money is the indicator of honesty.

### Results

A manipulation check showed that participants answering ‘why’ questions showed higher scores of abstraction (*M* = 3.08, *SD* = 0.63) than did those answering ‘how’ questions (*M* = -3.01, *SD* = 0.65) [*t*_(58)_ = 29.738, *p* < 0.001, *d* = 9.51]. The inter-judge agreement between two independent judges was satisfactory (*r* = 0.879, *p* < 0.001). With respect to the Stroop task, the mean RTs in incongruent trials and congruent trials were calculated. State self-control was indexed by the mean difference in RT between incongruent and congruent trials. Incorrect trials (<2.2%) were excluded from the analysis. As shown in **Table 2**, there were no significant differences in the mean RT in congruent trials of the Stroop task [*F*_(2,87)_ = 2.533, *p* = 0.085]. However, there was a main effect of condition on the mean RT in incongruent trials [*F*_(2,87)_ = 4.481, *p* = 0.014, $\eta_{p}^{2}$ = 0.093]. The mean RT in incongruent trials of the high-level construal condition was shorter than those of both the low-level construal [*t*_(87)_ = -2.487, *p* = 0.015, *d* = 0.64] and control [*t*_(87)_ = -2.687, *p* = 0.009, *d* = 0.65] conditions. More importantly, the self-control measure also differed among the three study conditions [*F*_(2,87)_ = 7.298, *p* = 0.001, $\eta_{p}^{2}$ = 0.144]. Follow up contrasts showed that participants under the high-level construal condition showed less Stroop interference (i.e., greater self-control) than did those under the low-level construal [*t*_(87)_ = -3.531, *p* = 0.001, *d* = 0.88] and control [*t*_(87)_ = -3.029, *p* = 0.003, *d* = 0.79] conditions. The connection between construal levels and state self-control was replicated in Experiment 2. In addition, the self-control measure did not differ between the low-level construal and control conditions [*t*_(87)_ = 0.503, *p* = 0.616].

**Table 2:** State self-control and the tendency toward moral behavior as a function of experimental conditions in Experiment 2.

Measure	Control		Low-level construal		High-level construal	
	*M*	*SD*	*M*	*SD*	*M*	*SD*
Mean RT in incongruent trials (ms)^a^	771.40	156.29	763.33	134.31	662.70	176.65
Mean RT in congruent trials (ms)	635.90	139.01	622.20	117.47	561.13	152.24
Self-control measure (ms)^a^	135.50	39.98	141.13	44.36	101.57	45.64
Money offered in the game (NT $0–160)^a^	46.17	19.86	49.83	15.84	61.50	13.53
Likelihood of returning undeserved money (%)^a^	50.0	9.1	46.7	9.1	76.7	7.7

^a^*These measures of the high-level condition were significantly different from those of the low-construal and control conditions, and there were no differences in these measures between the low-level construal and control conditions. Each condition involved 30 participants. The self-control measure was indexed by the mean difference in reaction time between incongruent and congruent trials in the Stroop task. Less Stroop interference indicates greater state self-control*.

**FIGURE 2:**
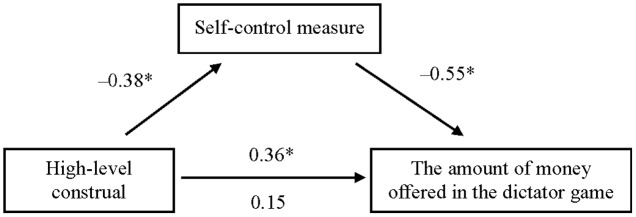
Mediation of the effect of exposure to “why” questions (high-level construal) regarding morality on the amount of money offered in the dictator game in Experiment 2. Values are standardized regression coefficients. The self-control measure was indexed as the mean difference in reaction time (ms) between incongruent and congruent trials in the Stroop task. Less Stroop interference indicates a state of greater self-control. On the lower path, the values below and above the arrow are the results of analyses in which the mediator was and was not included in the model, respectively. Asterisks indicate significant results (*p* < 0.01).

As predicted, the amount of money offered in a dictator game was associated with condition [*F*_(1,87)_ = 6.967, *p* = 0.002, $\eta_{p}^{2}$ = 0.138; **Table 2**]. Planned contrasts showed that participants under the high-level construal condition offered more money than did those under the low-level construal [*t*_(87)_ = 2.72, *p* = 0.008, *d* = 0.79] and control [*t*_(87)_ = 3.574, *p* = 0.001, *d* = 0.90] conditions. The amount of money offered did not differ between the low-level construal and control conditions [*t*_(87)_ = 0.855, *p* = 0.395]. Moreover, we examined whether state self-control mediated the link between the construal manipulation and the amount of money offered in the dictator game via linear regression. As the low-level construal and control groups did not differ in both the self-control measure and the amount of money offered, these two groups were combined as the reference group for the dummy variable (1 = high-level construal, 0 = low-level construal and control). A bootstrap analysis (Preacher and Hayes, 2004) showed that the indirect effect was significant (*B* = -7.65, *SE* = 2.31, 95% bias-corrected CI: 3.98–13.26; bootstrap resamples = 5000). Answering ‘why’ questions (high-level construal) predicted the self-control measure [*B* = -36.75, *SE* = 9.66, *t*_(88)_ = -3.804, *p* < 0.001], the self-control measure predicted the amount of money offered [*B* = -0.23, *SE* = 0.03, *t*_(88)_ = -7.248, *p* < 0.001], and the connection between high construal level and the amount of money offered [*B* = 13.50, *SE* = 3.71, *t*_(88)_ = 3.639, *p* < 0.001] was no longer significant [*B* = 5.73, *SE* = 3.36, *t*_(87)_ = 1.706, *p* = 0.092] when we controlled for the self-control measure (**Figure 2**). Thus, the mediation analysis results suggest that greater self-control, induced by construing morality at high levels, leads to an increased tendency toward altruism (less selfishness). We further tested an alternative model in which the amount of money offered in the dictator game was treated as the mediator of the association between the construal manipulation and state self-control. The dummy variable (1 = high-level construal, 0 = low-level construal and control) was used in the mediation analysis. When the amount of money offered was controlled for, the relationship between construing morality at high levels and less Stroop interference (i.e., greater self-control) was still significant [*B* = -17.40, *SE* = 8.70, *t*_(87)_ = -1.999, *p* = 0.048], suggesting that the amount of money offered was not a major mediator between the construal manipulation and state self-control.

Moreover, participants who offered more money in the dictator game were more likely to return undeserved money [1 = return, 0 = no return; *B* = 0.03, *SE* = 0.01, *p* = 0.019, Wald = 5.548, odds ratio (OR) = 1.03, 95% CI: 1.01–1.06], suggesting that these two dependent measures operate dependently. We found that the likelihood of returning underserved money was associated with condition [χ^2^_(2, *N* = 90)_ = 6.65. *p* = 0.036, Cramer’s *V* = 0.272; **Table 2**]. Two dummy variables (high-level construal vs. low-level construal; control vs. low-level construal) were created for our three study conditions, treating the low-level construal condition as the reference group. A logistic regression showed that participants under the high-level construal condition were more likely to return undeserved money (23 out of 30) than were those under the low-level construal condition (14 out of 30; *B* = 1.32, *SE* = 0.57, *p* = 0.019, Wald = 5.47, OR = 3.76, 95% CI: 1.24–11.39); however, no significant difference was observed between the control condition (15 out of 30; *B* = 0.13, *SE* = 0.52, *p* = 0.796) and the low-level construal condition with regard to the likelihood of returning underserved money. In addition, participants under the high-level construal condition were more likely to return undeserved money than were those under the control condition (*B* = 1.19, *SE* = 0.57, *p* = 0.035, Wald = 4.43, OR = 3.29, 95% CI: 1.09–9.95).

**FIGURE 3:**
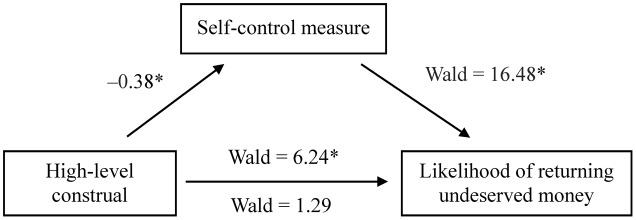
Mediation of the effect of exposure to “why” questions (high-level construal) regarding morality on the likelihood of returning undeserved money (1 = return, 0 = no return) in Experiment 2. The number of the path between high-level construal and self-control measure is a standardized regression coefficient. The self-control measure was indexed as the mean difference in reaction time (ms) between incongruent and congruent trials in the Stroop task. Less Stroop interference indicates a state of greater self-control. On the lower path, the values of Ward statistics below and above the arrow are the results of analyses in which the mediator was and was not included in the model, respectively. Asterisks indicate significant results (*p* < 0.05).

Furthermore, we employed linear regression and logistic regression to test whether state self-control mediated the link between the construal manipulation and honesty. The control and low-level construal groups were combined as the reference group for the dummy variable (1 = high-level construal, 0 = low-level construal and control). A high-level construal mindset predicted the self-control measure [*B* = -36.75, *SE* = 9.66, *t*_(88)_ = -3.804, *p* < 0.001], the self-control measure predicted the likelihood of returning undeserved money (*B* = -0.03, *SE* = 0.01, *Z* = -4.06, *p* < 0.001). The relationship between engagement in high-level construals and the likelihood of returning undeserved money (*B* = 1.26, *SE* = 0.50, *Z* = 2.497, *p* = 0.013) was no longer significant (*B* = 0.66, *SE* = 0.58, *Z* = 1.135, *p* = 0.256) after controlling for the self-control measure (**Figure 3**). A bootstrap analysis showed that the indirect effect was significant (*B* = 1.34, *SE* = 0.57, 95% bias-corrected CI: 0.46–2.55; bootstrap resamples = 5000). The results of mediation analysis suggest that greater self-control, induced by engaging in high-level construals toward morality, leads to increased honesty. In addition, an alternative model in which the likelihood of returning undeserved money was treated as a potential mediator of the link between the construal manipulation and state self-control was tested. The relationship between engagement in high-level construals and less Stroop interference remained significant [*B* = -24.21, *SE* = 8.79, *t*_(87)_ = -2.756, *p* = 0.007] after controlling for the likelihood of returning undeserved money, indicating that the honesty measure did not serve as a major mediator of the effect of construal levels on state self-control.

### Discussion

In short, Experiment 2 showed that construing morality at high levels led participants to act less selfishly (more altruistically) and increased their tendency toward honesty. The mediating role of state self-control in the connection between high-level construals and the tendency to perform moral behavior was replicated two times.

## GEneral Discussion

Based on recent advances in relation to the link between construal level and self-control (Fujita et al., 2006b; Fujita and Carnevale, 2012; Chiou et al., 2013; Chang and Chiou, 2015) and relating to the notion that self-control plays a crucial role in overriding selfish, antisocial impulses in favor of socially desirable behaviors (Baumeister and Exline, 1999; Hirschi, 2004; Mead et al., 2009; Chiou et al., in press), we hypothesized that a high-level construal mindset, relative to a low-level construal mindset, would be associated with greater self-control, thereby increasing the tendency to act morally. We found that answering “why” questions regarding morality (i.e., construing morality at high levels) promoted engagement in moral behavior, as reflected by a higher likelihood of helping (Experiment 1), a lower tendency toward selfishness (Experiment 2), and a greater likelihood of returning excess money (Experiment 2), compared with answering “how” questions regarding morality (i.e., construing morality at low levels). Furthermore, we showed that state self-control, as indexed by Stroop interference, mediated the connection between level of construal and morality-related behaviors. We provide the first demonstration showing that a brief construal-level intervention can engender a state of better self-control, leading people to perform ethical deeds.

Construal level theory posits that low-level construal enhances the cognitive accessibility of proximal temptations while overlooking distant goals, thereby undermining self-control (Fujita et al., 2006a; Fujita and Carnevale, 2012). Our findings indicated that greater self-control, as indexed by less Stroop interference, mediated the link between a high-level construal mindset and subsequent moral acts. Research has demonstrated that a concrete, low-level construal is associated with positive attitudes toward temptation, whereas an abstract, high-level construal is associated with negative attitudes (Fujita et al., 2006b; Fujita and Han, 2009). Given that the Stroop task requires self-control to over-ride the semantic meaning of a colored word, better self-control established by engaging in a high-level construal mindset was associated with less Stroop interference. Furthermore, the self-control model of morality assumes that humans are instinctively selfish and impulsive (Baumeister and Exline, 1999). To act morally, people need to exert self-control over selfish, antisocial impulses (Stevens and Hauser, 2004; Baumeister et al., 2007). Greater self-control has a crucial role in volunteering (Gottfredson and Hirschi, 1990). Moreover, honesty depends on self-control because it requires the exertion of self-control to forgo personal gains from dishonesty and act in a morally appropriate manner (Baumeister et al., 1994; Mead et al., 2009). Hence, participants construing morality at high (relative to low) levels were found to volunteer to code more data sheets, show less selfishness in the dictator game, and be more likely to return undeserved money. The strength model of self-control (Baumeister et al., 2007) may serve as a viable paradigm for understanding the observed connection between state self-control and our morality-related measures.

Recent studies have provided empirical evidence supporting the notion that prosocial behavior may arise from intuitive preferences rather than reflective, control processes (Zaki and Mitchell, 2013). For example, participants induced to thinking intuitively show increased cooperation (Rand et al., 2012). A reduced capacity to exert control was associated with increased cooperative and prosocial behavior (Cappelletti et al., 2011; Cornelissen et al., 2011; Rand et al., 2012). However, the experimental manipulations in these studies involved time pressure or distraction but not construal levels. Findings from extant research supporting the intuitive model of prosociality represent the effect of the intuitive versus the reflective thinking mode on prosocial decisions. In the current research, we employed a construal-level manipulation to induce a higher or lower level of state self-control. Our dependent measures (e.g., the opportunity to volunteer to help code data and the likelihood of returning undeserved money) differed from those used in the economic games employed by studies supporting intuitive prosociality (e.g., the ultimatum game of Cappelletti et al., 2011; the public goods game of Rand et al., 2012). Hence, the present findings indicate that moral engagement depends on the construal level and the degree of self-control.

As argued from the perspective of implementation intentions (Gollwitzer, 1999), forming implementation intentions refers to statements of the structure: “As soon as situation *x* occurs, I will perform behavior *y*.” The mechanism that is thought to be responsible for the effect of implementation intentions on performance of the behavior is the heightened accessibility of specified situational cues (Gollwitzer and Schaal, 1998; Gollwitzer, 1999). By forming an implementation intention, the intended behavior will be initiated automatically when the specified situational cue is encountered (Gollwitzer, 1993, 1999). According to the notion of CLT, the temporal distance would be longer as an event that is construed at abstract, high levels rather than concrete, low levels (Trope and Liberman, 2003; Liberman et al., 2007; Trope et al., 2007). Forming implementation intentions (i.e., concrete action plans) may increase the likelihood of performing the intended behavior rather than forming more abstract, general intentions (i.e., goal intentions that have the structure of “I intend to reach *x*!,” Gollwitzer, 1999). The observed association of construing morality at abstract, high levels and an increased tendency toward moral acts appears to be inconsistent with research regarding implementation intentions. However, our construal-level manipulation did not ask participants to form implementation intentions (concrete action plans). We only had participants construe morality at abstract or concrete levels. In addition, participants did not aware the connection between the construal-level manipulation task and the morality-related measures. The effect of implementation intentions (versus goal intentions or no-implementation intentions) on the likelihood of performing actions and events is driven by the heightened accessibility of specified situational cues (see Gollwitzer, 1999, for a related review) and the temporal distance of activity enactment (Liberman et al., 2007; Trope et al., 2007; Trope and Liberman, 2010). Two key differences between implementation intention studies and the present research are that the latter examined the effect of construal level on state self-control and demonstrated the mediating role of state self-control in the link between construing morality at high versus low levels and the tendency to perform moral acts.

The present findings contribute to the literature in several important ways. First, we provide experimental evidence that a mindset-based manipulation of construal level is sufficient to encourage moral behavior. Reminding people of codes of ethics, restricting anonymity, and increasing people’s motivation to maintain a positive moral outlook have been identified as effective interventions to promote moral behavior (Ayal et al., 2015). The current research supplements the literature with an innovative strategy for revising people’s unethical behavior. Second, our findings supplement the literature on the relationship between degree of self-control and engagement in moral behavior (Baumeister and Exline, 1999; DeWall et al., 2008; Mead et al., 2009; Steinbeis et al., 2012). Finally, construing morality at high levels may engender a state of better self-control and, thereby, inhibit the impulse to engage in unethical acts. The present study indicates that the level at which morality is construed may be more closely connected to moral engagement than previously believed.

We acknowledge that our construal-level manipulation was limited to a dichotomous variable. Employing only the why/how paradigm to manipulate construal level may have led to mono-operation bias and limited the generalizability of the findings. In this research, participants were encouraged to focus on the “why” (high-level construal) or “how” (low-level construal) aspects of morality. Whether a high-level construal (relative to a low-level construal) mindset toward morality-unrelated contents would be sufficient to produce similar effects remains unanswered. Future research should employ construal levels (high vs. low) using a construal focus (moral vs. neutral) factorial design to test this possibility. Moreover, the judges rated only the abstractness of participants’ answers to the manipulation check. The lack of ratings of the relatedness to morality did not allow us to examine whether activation of the moral domain was the same across our experimental conditions. We employed the Stroop task to measure state self-control. Alternative self-control measures, such as the operation span task (e.g., Turner and Engle, 1989; Healey et al., 2011; Lurquin et al., 2016), should be used for convergent validation. Furthermore, according to Cohen’s (1988) benchmarks of effect size, the effect of construal level on state self-control, as indexed by Stroop interference, was strong. However, Experiment 2 showed that the difference in Stroop interference between the low-level construal and control conditions was not significant, suggesting that construing morality at low levels does not consume self-control strength as expected. Although the current research indicates a connection between a high-level construal mindset of morality and a greater tendency toward moral behavior, our findings represent the immediate effects of high/low construal levels on moral acts in a laboratory setting. Caution should be exercised when generalizing our results to naturalistic settings. The long-term effect of high-level construal on increased morality needs further examination with a longitudinal design. Additionally, future research may examine whether a high-level construal intervention could also be effective in the promotion of other ethical behaviors such as charitable donation, cooperation, and sacrifice behavior.

## Conclusion

The current research indicates that a state of greater self-control induced by construing morality at high levels is associated with a stronger tendency to act morally. The present findings indicate that construing morality at high versus low levels can lead individuals to exert better control over selfish, unethical impulses, as reflected by a higher likelihood of acting morally. Therefore, people may diligently monitor whether a high-level construal mindset is related to moral engagement in everyday life. Given that high-level construals appear to enhance prospective self-control (Rogers and Bazerman, 2008; Fujita and Roberts, 2010), people may construe morality at high levels (e.g., maintain a positive self-image; earn the respect of others; achieve mutual benefits from each other) to achieve better control over the temptation to profit from immoral behavior. Interventions and polices that remind people to adopt high-level construal in relation to moral regulation may promote moral engagement. In this way, thinking abstractly about morality may constitute a way of acting morally.

## Ethics Statement

This research was carried out in accordance with the ethical standards of American Psychological Association. The research protocol was approved by the research ethical committee of the Kaohsiung Medical University. The participants signed an informed consent form stating that the duration of the study and explaining they could withdraw from the study, and that the data would be anonymous.

## Author Contributions

C-CW, W-HW, and W-BC designed the study described in the manuscript and supervised the data collection. W-HW and W-BC participated in the data collection. C-CW and W-BC performed the analyses. C-CW and W-BC wrote the first draft of the paper. The first draft of the paper was revised by W-HW.

## Conflict of Interest Statement

The authors declare that the research was conducted in the absence of any commercial or financial relationships that could be construed as a potential conflict of interest.
